# Exposure profiles in pregnant women from a birth cohort in a highly contaminated area of southern Italy

**DOI:** 10.1038/s41598-023-41865-0

**Published:** 2023-09-08

**Authors:** Gaspare Drago, Silvia Ruggieri, Mario Sprovieri, Giulia Rizzo, Paolo Colombo, Cristina Giosuè, Enza Quinci, Anna Traina, Amalia Gastaldelli, Fabio Cibella, Simona Panunzi

**Affiliations:** 1 Institute for Biomedical Research and Innovation, National Research Council of Italy, Via Ugo La Malfa 153, 90146 Palermo, Italy; 2 Institute of Marine Sciences, National Research Council of Italy, Arsenale-Tesa 104, Castello 2737/F, 30122 Venice, Italy; 3 Institute of Anthropic Impacts and Sustainability in Marine Environment, National Research Council of Italy, Lungomare Cristoforo Colombo 4521, 90149 Palermo, Italy; 4 Institute of Anthropic Impacts and Sustainability in Marine Environment, National Research Council of Italy, Via del Mare 3, Torretta Granitola, 91021 Trapani, Italy; 5 Institute of Clinical Physiology, National Research Council of Italy, Via Giuseppe Moruzzi 1, 56124 Pisa, Italy; 6 Institute for System Analysis and Computer Science-BioMatLab, National Research Council of Italy, Via dei Taurini 19, 00168 Rome, Italy

**Keywords:** Public health, Epidemiology

## Abstract

Protecting the health of pregnant women from environmental stressors is crucial for reducing the burden of non-communicable diseases. In industrially contaminated sites, this action is particularly challenging due to the heterogeneous pollutant mixtures in environmental matrices. The aim of this study was to evaluate distribution patterns of mercury, hexachlorobenzene and polychlorobiphenyls in the serum of 161 pregnant women recruited in the framework of the Neonatal Environment and Health Outcomes (NEHO) cohort and living both inside and outside the National Priority Contaminated Site (NPCS) of Priolo. Food macro-categories were determined, and serum levels of contaminants were used to perform k-means cluster analysis and identify the role of food in pollutant transfer from the environment. Two groups of mothers with high and low measured pollutant levels were distinguished. Concentrations in mothers in the high-exposure cluster were at least twofold for all the evaluated pollutants (p < 0.0001) and included mothers living inside and outside NPCS, with a predominance of individuals from the NPCS (p = 0.045). Fish consumption was higher in the high-exposure cluster (p = 0.019). These findings suggest a link between contamination of environmental matrices such as sediment with maternal exposure, through the intake of local food. Such consideration appears poorly investigated in the context of contaminated sites.

## Introduction

A mounting body of evidence has convincingly linked the aetiology of several non-communicable diseases to environmental stressors. There is, therefore, an increasing need to prioritise strategies of environmental protection in order to significantly reduce the burden of chronic diseases^1^. In this perspective, the European Commission has published an ambitious agenda to challenge the effects of environmental degradation by 2050^2^. A parallel approach seeks to reduce the health consequences of environmental deterioration and points to identifying human exposure pathways in order to (i) protect health by preventing/reducing the detrimental effects of hazardous agents, and (ii) plan specific regulatory measures which can lead to improved environmental conditions^3^. Both these lines of action are even more demanding in areas characterised by highly-complex industrial contamination where socio-economic progress, environmental pollution and impacts on health are deeply interconnected^4^. People living near such contaminated areas are exposed to multiple and significant health threats. In fact, the simultaneous exposure to mixtures of several environmental pollutants—even at low concentrations—may produce a risk different from that produced by single contaminant exposure^5^. Moreover, women of childbearing age and developing foetuses are highly susceptible subgroups which are an absolute priority for safeguarding future generations^6–8^. Exposure to contaminants that occurs during these ‘time windows’ may have long-term deleterious consequences during early infancy and may generate risks for major diseases later in life^9,10^.

Priolo is a large industrialised marine-coastal area in southern Italy, hosting one of the largest European petrochemical plants. It covers an area of 550 km^2^ with a dense concentration of refineries, petrochemical and cement plants and waste dumps^11^. Within this area, Augusta Bay (the coastal-marine area of ~ 25 km^2^ bordering the eastern boundary of the National Priority Contaminated Site—NPCS) was a main source of mercury (Hg) and other priority organic compounds discharged at sea until the 1970s as a by-product of a chloro-alkali plant and other petrochemical factories^12–14^. In the same area, several studies have measured significant contamination by polychlorinated biphenyls (PCBs), hexachlorobenzene (HCB) and other classes of emerging pollutants detected in both environmental compartments and biota^15–18^. Mercury and persistent organic pollutants (POPs) are long-lasting in the environment and bioaccumulate/biomagnify in tissues of living organisms. Foetal exposure to these chemicals during pregnancy can be a critical factor in a wide range of disorders later in life^19–23^. In this light, human biomonitoring constitutes a powerful way to understand the specific ways in which pregnant women may come into contact with such contaminants and to investigate how pollutant content in the organism varies following the dynamic of multiple exposure sources^24,25^. This study is primarily aimed at evaluating the levels and distribution patterns of a few classes of pollutants—selected among some of those that specifically characterise the study area—in serum samples collected from pregnant women living in the Priolo area and enrolled in the Neonatal Environment and Health Outcomes (NEHO) birth cohort^26^. The study aimed at exploring the exposure profiles of a sample of pregnant women from the Priolo area through the analysis of a mixture of pollutants, specifically Hg, HCB and three highly chlorinated PCB congeners with long biological half-lives (PCB138, PCB153 and PCB180). These specific contaminants had already been reported in several environmental compartments at relatively high concentration levels. This should offer crucial clues regarding the exposure mechanisms and pathways through which mixtures of environmental pollutants may affect such a critical population group.

## Methods

### Study sample

Between January 2018 and January 2020, the NEHO cohort enrolled 561 pregnant women living in the NPCS of Priolo and in Local Reference Areas (LRAs) located outside the NPCS boundaries (see Supplementary Material, Table A1) but characterised by similar socio-demographic features. For the present study, 161 women were randomly selected from the NEHO birth cohort^27^, 85 residing in the NPCS and 76 residing in LRAs: no significant difference was found between the selected sample and the whole birth cohort in Priolo area, with the exception of the numbers of previous pregnancies (Supplementary Material, Table A2). Briefly, after reading a detailed information sheet, all the participants, during their last trimester of pregnancy, were required to sign a consent form confirming their understanding of the project’s aim. Detailed information was collected from the mothers using web-based questionnaires at enrolment.

The study was approved by the Ethics Committee “Catania 2” (July 11, 2017, No. 38/2017/CECT2). All procedures were conducted following the Declaration of Helsinki. The adopted protocol was compliant with the General Data Protection Regulation (UE 2016/679) and Italian data protection laws.

### Questionnaire

The present study combines a variety of information from a subset of questionnaires with the aim of shedding light on possible associations between lifestyle and detected concentrations of pollutants in serum. Mothers enrolled in the NEHO cohort were asked to fill in different questionnaires. The “Baseline—first part” questionnaire provided information on maternal health and lifestyle during the gestational period. Some of the questions were aimed at retrieving information about the consumption habits of different types of food.

The maternal characteristics and socio-environmental factors were age, body mass index before pregnancy (BMI), marital status, weeks of gestation and educational level, which originally was categorised into four levels: “Elementary school”, “Middle school”, “High school”, “Degree or higher qualification”. Here, educational levels were categorized on the basis of the years of education: “Elementary school” and “Middle school” were unified into “0–8 years of education”; while “High School” and “degree or higher qualification” were considered as “8–13 years of education” and “more than 13 years of education” respectively. Because the present study is also focused on the frequency of food consumption, the original questionnaire items collecting information about the consumption frequencies of the considered categories were modified as follows: “Never” was recorded in 0 days/month; “Once per month” was recorded in 1 day/month, “2/3 times per month” was recorded in 2.5 days/month; “Once per week” was recorded in 4 days/month; “2/3 times per week” was recorded in 10 days/month; “2/3 times per week” was recorded in 18 days/month; “Every day” was recorded in 30 days/month. A standard portion of each food was then considered in order to compute the total amount (in grams) consumed by each mother in one month. According to the National Recommended Energy and Nutrient Intake Levels^28^, a standard fish portion corresponds to 150 g; 100 g is the standard portion for each type of meat; dairy products were expressed as a sum of yogurt 125 g, milk 125 g and fresh cheese 50 g; 200 g were used for eggs and vegetables, except for leafy and stem vegetables, for which a standard portion of 80 g was assumed. The quantity of vegetables consumed was computed as the sum of stem vegetables, leafy vegetables, Brassicaceae, raw and cooked vegetables.

In addition, to build a logistic regression model analysis, levels of consumption were rearranged to obtain a homogeneous distribution among categories of fish and vegetable consumption. To this aim, fish consumption was categorized into three levels: 0 if no consumption, 1 if only one time in a month, and 2 if equal to or more than 2/3 times in a month. As concerns vegetables, the classification was performed by unifying “no” and “low” (only one time in a month) consumption into level 0; level 1 referred to a consumption from 2/3 in a month to one time per week; level 2 was assigned to mothers with a “greater” (equal to or more than 2/3 times per week) consumption. The different classifications adopted for fish and vegetables were due to the different frequencies of each food item (i.e., while “no consumption” class for fish presented a sufficient recurrence, the same class for vegetables was almost absent).

### Analytical procedure

Maternal blood samples were collected during the last trimester of pregnancy, and serum was then separated by centrifugation and temporarily stored at − 20 °C in each maternal unit before being transported on dry ice to the NEHO biobank for long-term storage at − 80 °C until the analysis was carried out. Analyses of POPs (HCB and three congeners of PCBs 138, 153 and 180) in maternal serum were performed at the National Institute for Health and Welfare, Chemical Exposure Unit, Kuopio, Finland, with an Agilent 7000B gas chromatograph triple quadrupole mass spectrometer (GC–MS/MS). Ethanol and ^13^C-labelled internal standards were added to samples. Dichloromethane-hexane was added for extraction, followed by the addition of activated silica gel to bind the sample water and ethanol. The dichloromethane-hexane layer was poured into a solid phase extraction cartridge (SPE cartridge) containing 44% sulphuric acid silica, 10% silver nitrate impregnated silica and a mixture of sodium sulfate and silica. The lower semi-solid layer was extracted again with dichloromethane-hexane that was also poured into an SPE-cartridge. Elution of the SPE-cartridge was continued with dichloromethane-hexane, and the eluate was concentrated for GC–MS/MS. The quantification was performed by multiple reaction monitoring using an Agilent 7890A gas chromatograph/Agilent 7010 triple quadrupole mass spectrometer with DB-5MS UI column (J&W Scientific, 20 m, lD 0.18 mm, 0.18 µm). Reference materials for organic contaminants in human serum were analysed to estimate accuracy (SRM 1589a—National Institute of Standards and Technology, Gaithersburg, MD, USA). Recoveries ranged between 96 and 104% for each PCB and HCB analyte. Analytical precision was routinely better than 3% RSD%.

Total serum triglycerides and cholesterol concentrations were assayed by certified spectrophotometric methods (Randox Laboratories, Crumlin, UK) at the Institute of Clinical Physiology of the National Research Council, Pisa, Italy. Total lipids were formulated according to the following equation^29^:

$${\text{Total}}\;{\text{lipids}}\;({\text{mg/dL}}) \, = \, 1.12 \times {\text{total}}\;{\text{cholesterol}} + 1.33 \times {\text{triglycerides}} + 148.$$

Lipid-normalized organochlorine concentrations were calculated from wet weight concentrations divided by total lipids and expressed as ng/g of total lipids.

Analyses of Hg were performed at the laboratory of LERES (Laboratoire d'Etude et de Recherche en Environnement et Santé) at the French School of Public Health—EHESP (Rennes, France), following the procedures described by Davies et al.^30^. The 161 serum samples were analysed by a plasma torch coupled with tandem mass spectrometer (ICP-MSMS 8800, Agilent Technologies) after a mineralisation step by adding nitric acid and heated with a heating block (Hotblock Pro, model SC-189, Environmental Express) at 83 °C for 4 h. Matrix effects correction was guaranteed through the use of internal standards (Sc, Ge, 77Se, Rh, Re and Ir). All internal standards were quantified in samples with less than 25% variation. Certified or internal control materials (measured additions) of blood and serum were added to the series (Utak level 1, Seronorm level 1) in order to guarantee the smooth running of the different stages and to cover the set of blood matrices. The results were validated since the concentrations of the controls were located within the limits of the control charts. This procedure was accredited by the French accreditation committee (CoFrac) in January 2020.

Concentrations below the limit of quantification (LOQ) were replaced by LOQ/2.

### Statistics

Women were grouped according to their pollutant serum levels. A non-supervised k-means algorithm was used on the scaled logarithms to base 2. Concentrations were log-transformed to obtain a normal distribution and then standardized to define the concentrations on the same scale. The Shapiro–Wilk test was used to determine whether the variables came from a normal distribution. The optimal number of clusters was estimated by computing both the Within Cluster Sums of Squares (WCSS), for the Elbow method, and the average silhouette.

The classification into clusters was used as a factor for testing associations with the NPCS and LRAs, as well as with other relevant qualitative variables using a Chi-Squared test or a Fisher exact test, when appropriate. The dependence of quantitative variables on the individuated clusters and the possible association between the clusters and the quantities of food consumed were assessed by means of a Mann–Whitney *U* test.

Aimed at identifying the variables to be introduced in a multivariable model, univariable logistic regression models were used to test the dependence of cluster on the consumption of each food category and on socio-demographic predictors. Only predictors significant at a p level of 10% in the univariable analysis were then included into a multivariable logistic model in order to limit the number of predictors given the small size of the study sample.

To assess the contribution of dietary habits and socio-demographic characteristics, two multivariable models were implemented: one including only food items, and the other one including socio-demographic variables (i.e., maternal age, area of residence and educational level). A further model including both types of variables was also built. In addition to the above described procedure, a LASSO regression including all the food categories and the socio-demographic predictors was also performed by means of the IsLASSO R package^31^ and result compared.

Moreover, a Weighted Quantile Sum (WQS) regression^32,33^ was performed to assess the impact of food consumption in relation to exposure clustering, including only the predictors significant in the univariable logistic models. The repeated holdout procedure^34^, with 100 repetitions, was used to stabilize results. For each repetition 100 bootstraps were implemented for a total of 10,000 estimates. A 30%/70% training/testing splits was used.

A heatmap was used to graphically show the representative levels of blood pollutants in each cluster. p-values < 0.05 were considered significant. All the analyses were performed in R, version 4.1.3^35^.

### Ethics approval

The NEHO study protocol has been approved by the Ethics Committee “Catania 2” for the NPCS of Priolo (11 July 2017, n. 38/2017/CECT2), and strictly followed the Declaration of Helsinki. Each participant read the information sheet and signed the informed consent. The participant information sheet is available at the NEHO website (http://www.neho.it). All the adopted procedures were compliant with the General Data Protection Regulation (UE 2016/679) and Italian laws concerning data protection.

## Results

### Study sample characteristics

Table 1 reports a description of the enrolled women with relevant demographic and socio-economic traits, separately by residence (LRAs or NPCS). Mean age (± SD) was 30.7 ± 4.7 years, with no difference between the two groups (p = 0.811). Similarly, BMI and the variable “weeks of gestation” emerged as not statistically different. The association between educational level and location was significant (p = 0.023), highlighting a larger percentage of mothers with a higher educational level living in the NPCS. Marital status (married, never married/separated) was not significantly different (p = 0.800), with the highest percentage of married women (65.0%) reflecting the distribution observed in the whole NEHO cohort^36^. Table 2 reports the pollutant concentrations in maternal serum of residents in LRAs and NPCS. Pollutants in serum of women living in the highly contaminated area were significantly higher than those detected in samples from the reference areas, excluding Hg (p = 0.402).

**Table 1:** Socio-demographic characteristics of the study population.

	Total (N = 161)	LRA (N = 76)	NPCS (N = 85)	p value
	Mean (± SD)	Mean (± SD)	Mean (± SD)	
Age (years)	30.71 (± 4.66)	30.64 (± 4.87)	30.78 (**± **4.50)	0.811*
BMI (kg/m^2^)	23.24 (± 4.78)	23.11(± 4.95)	23.35 (**± **4.65)	0.550*
Gestational length (weeks)	39.04 (± 1.22)	38.91(± 1.17)	39.16(**± **1.26)	0.223*

	N total	N (%)	N (%)	p value
Educational level				**0.023**^#^
Secondary school or lower qualification	30	19 (63.3%)	11 (36.7%)	
High School	92	35 (38.0%)	57 (62.0%)	
Degree or higher qualification	39	22 (56.4%)	17 (43.6%)	
Marital status				0.800^#^
Married	104	49 (47.1%)	55 (52.9%)	
Never married/Separated	55	26 (47.3%)	29 (52.7%)	
Missing	1	1	0	
Previous pregnancy				0.222^#^
Nulliparous	77	39 (50.6%)	38 (49.4%)	
Parous	76	30 (39.5%)	46 (60.5%)	
Missing	8	7	1	
Dental amalgams				0.391^#^
Yes	85	36 (42.4%)	49 (57.6%)	
No	69	34 (49.3%)	35 (50.7%)	
Missing	7	6	1	

*LRA* local reference area, *NPCS* national priority contaminated site, *SD* standard deviation; *p-value from Mann–Whitney *U* test for the differences between the quantitative variables and the residence areas; ^#^p-value from a Chi-square test for the differences between the qualitative variables and the residence areas. Significant values are in bold.

**Table 2:** Maternal serum contaminant levels. Serum levels of HCB and PCBs were normalized to total lipid content and reported in ng/g lipids.

Pollutant	LOQ	N (%> LOQ)	Total (N = 161)		LRA (N = 76)			NPCS (N = 85)			Median (IQR)	p value*
			Mean (SD)	Geom. mean (Min–Max)	Median (IQR)	Mean (SD)	Geom. Mean (Min–Max)	Median (IQR)	Mean (SD)	Geom. Mean (Min–Max)		
Hg (µg/L)	0.40	111 (68.9%)	0.84 (0.75)	0.59 (0.2–4.56)	0.59 (0.2–1.21)	0.80 (0.75)	0.55 (0.2–4.56)	0.57 (0.2–1.17)	0.88 (0.76)	0.62 (0.2–3.56)	0.67 (0.2–1.22)	0.402
HCB (ng/g)	10.00 (ng/L)	161 (100%)	8.47 (6.19)	7.39 (2.24–57.43)	7.13 (5.55–9.79)	7.89 (6.53)	6.80 (2.24–57.43)	6.78 (5.07–9.07)	8.99 (5.85)	7.97 (2.77–43.03)	7.18 (6.05–10.43)	**0.041**
PCB138 (ng/g)	5.00 (ng/L)	161 (100%)	9.88 (8.64)	8.02 (1.59–84.03)	7.78 (5.18–11.62)	8.60 (6.78)	7.07 (1.59–51.76)	7.09 (4.76–10.77)	11.02 (9.93)	8.97 (2.55–84.03)	8.97 (6.30–12.64)	**0.022**
PCB153 (ng/g)	5.00 (ng/L)	161 (100%)	17.48 (15.67)	13.97 (2.18–140.38)	13.75 (9.17–21.41)	15.23 (13.25)	12.19 (2.18–103.45)	11.80 (7.89–19.22)	19.50 (17.39)	15.77 (4.38–140.38)	15.28 (10.58–22.53)	**0.016**
PCB180 (ng/g)	5.00 (ng/L)	161 (100%)	12.90 (12.10)	9.84 (1.07–91.27)	9.89 (6.11–16.09)	11.20 (10.73)	8.45 (1.07–80.56)	8.08 (5.08–13.79)	14.42 (13.08)	11.28 (2.96–91.27)	11.33 (7.04–16.98)	**0.021**
ΣPCB^a^ (ng/g)	–	–	40.26 (35.98)	32.02 (4.84–315.67)	31.52 (23.89–51.92)	35.03 (30.52)	27.88 (4.84–235.78)	26.58 (17.62–44.10)	44.93 (39.84)	36.24 (9.88–315.67)	34.69 (23.89–51.92)	**0.017**

*LRA* local reference area, *NPCS* national priority contaminated site, *LOQ* limit of quantification, *SD* standard deviation, *IQR* interquartile range; *p-values from Mann–Whitney *U* test for the differences in pollutant levels between residence areas; ΣPCB^a^: Sum of PCB138, PCB153 and PCB180 congeners; concentrations below the LOQ were replaced by LOQ/2 before lipid adjustment. Significant values are in bold.

### K-means clustering

**Figure 1:**
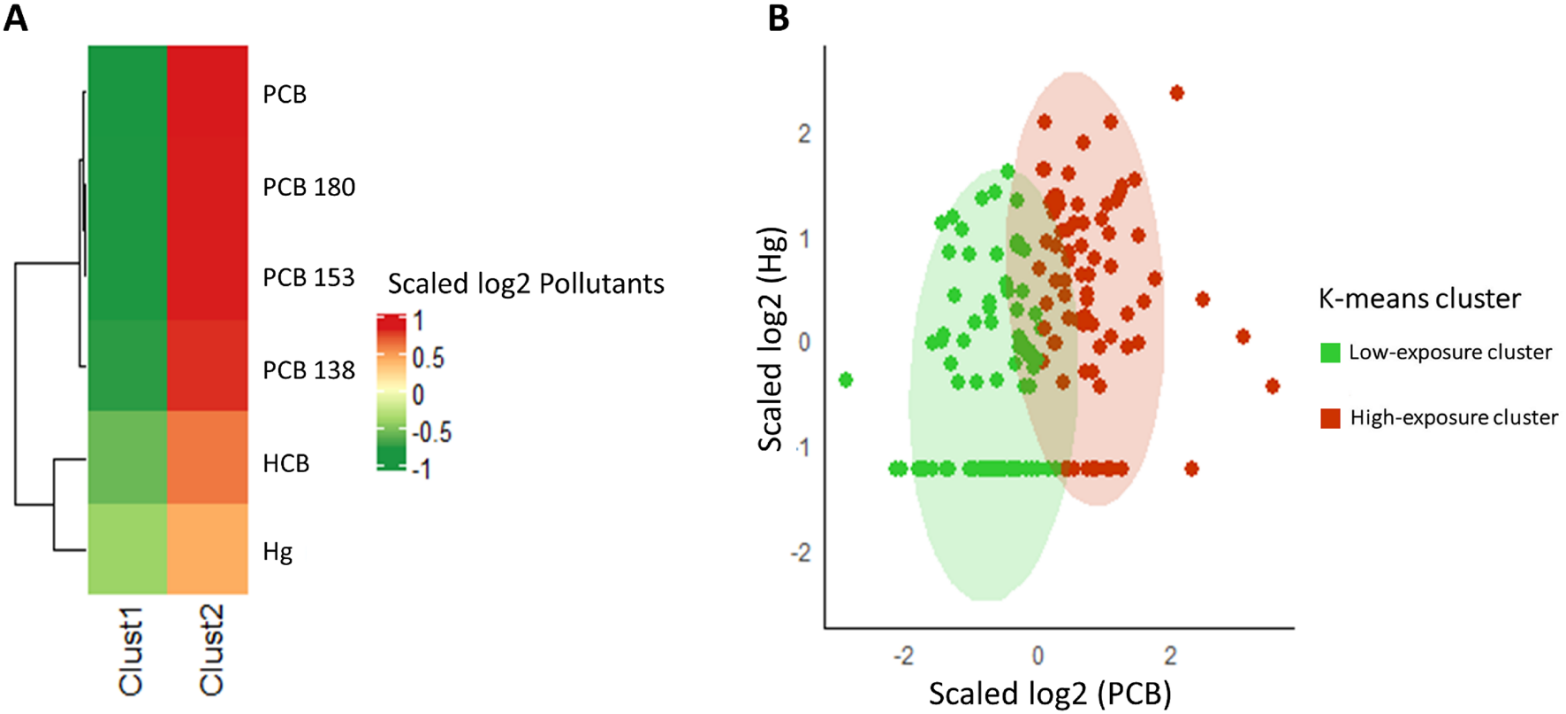
(**A**) Heatmap generated by k-means clustering analysis; (**B**) Groups of individuals in the two clusters identified according to the levels of Hg and sum of the PCBs. Points aligned at the bottom of the figure represent the non-quantifiable values (i.e., values below the LOQ).

From the k-means cluster analysis, both the Elbow and Silhouette methods identified 2 as the optimum number of clusters. Figure A1 (panels A and B in Supplementary Material) shows the values of the two indices in correspondence with different Ks. The k-means procedure subdivided the sample into high-exposure (H-Exp) and low-exposure (L-Exp) groups. The heatmap in Fig. 1 shows the average values of the pollutants in the two clusters (Panel A). Panel B shows how individuals were grouped into the two clusters based on the concentration levels of Hg, HCB and PCBs. Table A3 in the Supplementary Materials reports the measured pollutants from the two clusters highlighting the significant differences for all the pollutants measured in serum. Table 3 shows the mothers’ distribution in the two clusters by their socio-economic traits. In particular, the association between clusters and area of residence (NPCS vs LRA) was significant (p = 0.045), with the largest percentage of women living in the NPCS belonging to the H-Exp cluster (47 of 77—61%). In addition, individuals from this latter cluster were older than mothers with lower levels of contaminants (p < 0.001) and with higher educational levels (p = 0.018). The role of food consumption as a driver of contamination to mothers was investigated by means of the Mann–Whitney *U* test. Among the considered food categories (including meat, milk, eggs, fish and vegetables), consumption of fish and vegetables was significantly higher in the H-Exp cluster than in the L-Exp Cluster (p = 0.019 and p = 0.017 respectively, Table 4).

**Table 3:** Socio-demographic characteristics of the two clusters.

	Total (N = 161)	Cluster 1 (N = 84) Low exposure	Cluster 2 (N = 77) High exposure	p value
	Mean (± SD)	Mean (± SD)	Mean (± SD)	
Age (years)	31.3 (± 4.7)	29.3 (± 4.3)	33.5 (± 4.1)	< 0.0001*
BMI (Kg/m^2^)	23.2 (± 4.8)	23.5 (± 5.4)	22.9 (± 4.0)	0.834*
Gestational length (weeks)	39.0 (± 1.2)	39.1 (± 1.2)	40.0 (± 1.3)	0.650*

	N total	N (%)	N (%)	p value
Residence area				**0.045**^#^
NPCS	85	38 (44.7%)	47 (55.3%)	
LRA	76	46 (60.5%)	30 (39.5%)	
Educational level				**0.018**^**#**^
Secondary school or lower qualification	30	21 (70.0%)	9 (30.0%)	
High School	92	49 (53.3%)	43 (46.7%)	
Degree or higher qualification	39	14 (35.9%)	25 (64.1%)	
Marital status				0.388^#^
Married	104	52 (50.0%)	52 (50.0%)	
Never married/Separated	56	32 (57.1%)	24 (42.9%)	
Missing	1	0	1	
Previous pregnancy				0.572^#^
Nulliparous	77	41 (52.6%)	37 (47.4%)	
Parous	76	36 (48.0%)	39 (52.0%)	
Missing	8	7	1	
Dental amalgams				0.323^#^
Yes	85	40 (47.1%)	45 (52.9%)	
No	69	38 (55.1%)	31(44.9%)	
Missing	7	6	1	

*LRA* local reference area, *NPCS* national priority contaminated site, *SD* standard deviation; *p-value from Mann–Whitney *U* test for the differences between the quantitative variables and the two clusters. ^#^p-value from Chi-square test for the differences between the qualitative variables and the two clusters. Significant values are in bold.

**Table 4:** Consumption of food in g/month in the two clusters, identified by the k-means procedure.

	Cluster 1 (N = 84) Low exposure		Cluster 2 (N = 77) High exposure		p value*
	Mean (SD)	Median (25–75%)	Mean (SD)	Median (25–75%)	
Total fish	708.3 (784.8)	450 (150–1125)	929.4 (802.4)	600 (450–1312.5)	**0.019**
Total meat	1392.7 (1174.4)	1000 (400–1912.5)	1303.2 (1167.7)	900 (450–1600)	0.800
Dairy products	3021.3 (2080.8)	3725 (1393.75–4500)	3314.3 (2097.8)	3750 (1450–5200)	0.377
Eggs	894 (558.8)	800 (800–800)	911.7 (509.9)	800 (800–800)	0.804
Vegetables	4657.4 (2476.4)	4400 (3160–5480)	5453.2 (2820)	5440 (3800–6400)	**0.017**

*p-values computed for the differences between the two clusters by Mann–Whitney *U* test. Significant values are in bold.

**Figure 2:**
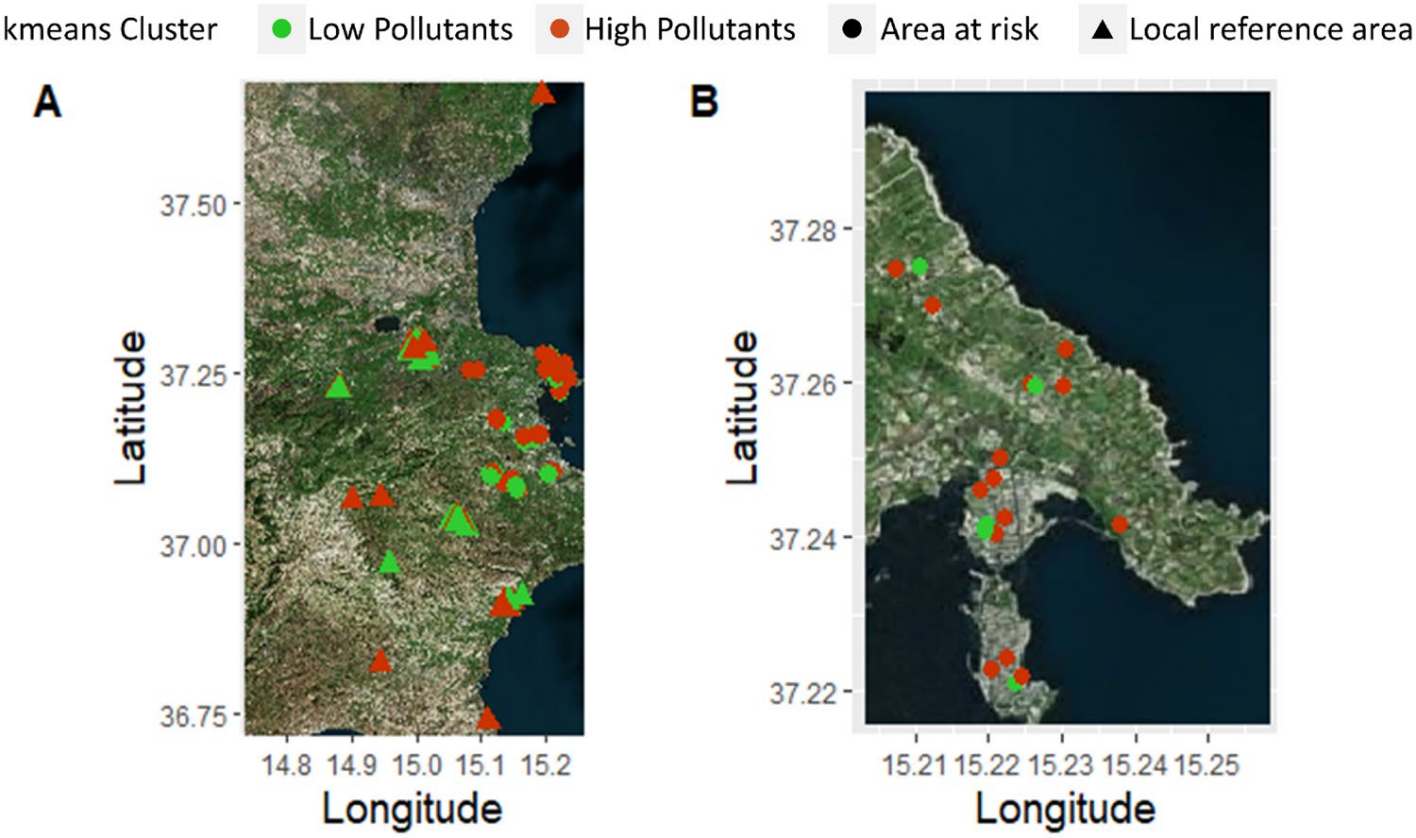
Geographical distribution of mothers in the Priolo area according to the k-means clustering (low vs high pollutant levels) and area of residence (NPCS vs LRA). (**A**) Refers to the entire study area. (**B**) Refers to the municipality of Augusta and to Augusta Bay. The maps were created using the OpenStreetMap package (https://cran.r-project.org/package=OpenStreetMap) of R version 4.1.3.

Figure 2 shows the geographical distribution of mothers in the Priolo area according to the k-means clustering (H-Exp and L-Exp) and their area of residence (LRAs vs NPCS). Residences of the mothers from the H-Exp cluster are shown in red, while those from the L-Exp cluster are in green. Moreover, circles and triangles discriminate between the mothers residing in the NPCS and LRA, respectively. In the municipality of Augusta, within the NPCS, most of the mothers were associated with the H-Exp cluster (Fig. 2B).

**Figure 3:**
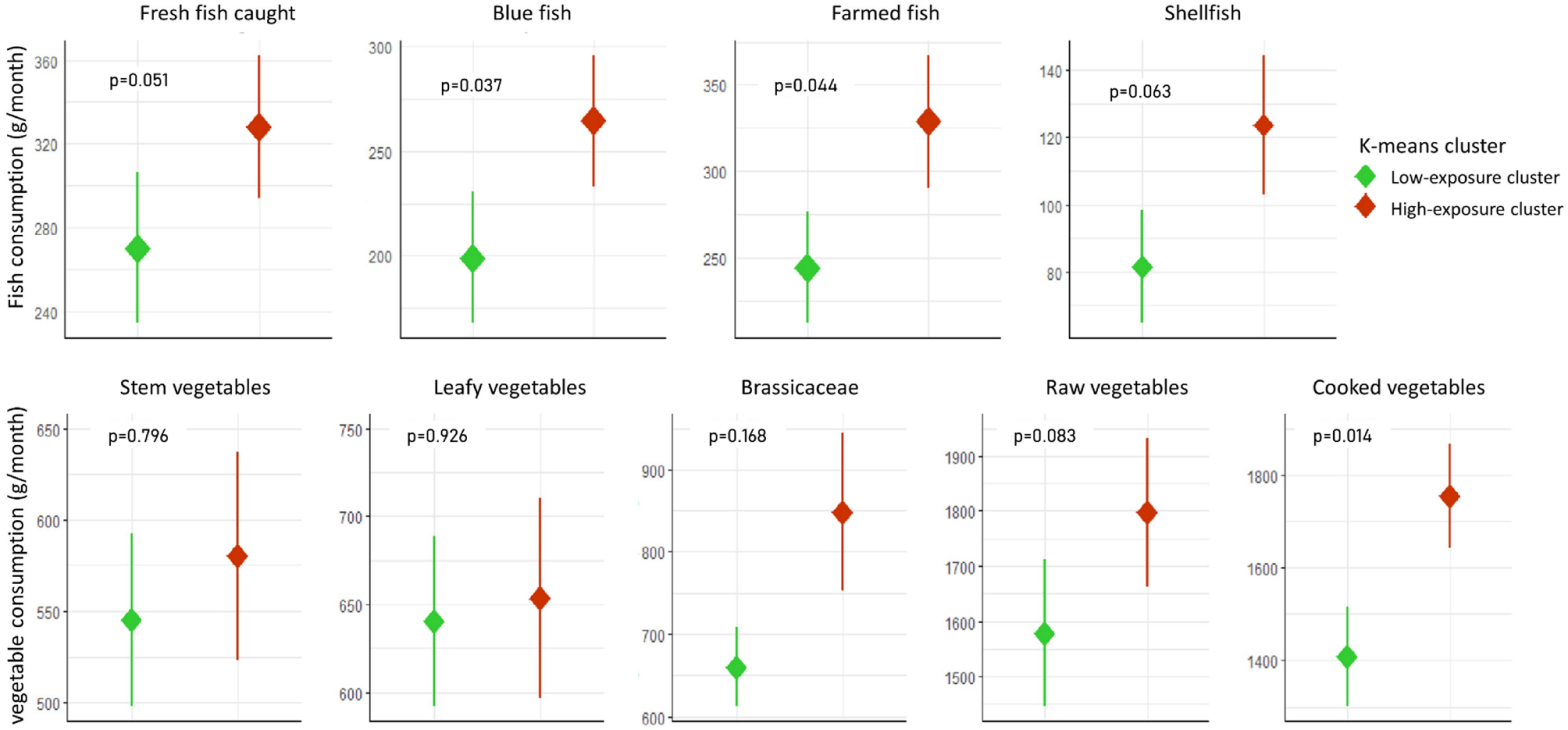
Average of fish (upper panel) and vegetable (lower panel) consumption in the two clusters. The rhombus, indicates the average consumption in each clusters, the bar indicates the standard error. p-values from Mann–Whitney *U*-test.

Specifically, Fig. 3 (upper panel), shows the values of the average consumption of the categories of fish considered in the two k-means clusters (High- and Low-exposure levels). The p values from the Mann–Whitney *U* test are also reported in the corresponding graphs. The average consumption of “Fresh caught fish”, “Blue fish” and “Farmed fish” resulted significantly different in the two clusters, with the women belonging to the H-Exp cluster consuming larger quantities of fish. Differently, “Shellfish” consumption was not significantly different between the two groups, also considering that shellfish are barely present in the diet of all the individuals studied. The average consumption of cooked vegetables was significantly higher in women belonging to the H-Exp cluster (p = 0.014). Notably, while mothers of the H-Exp cluster preferentially consumed fish of local origin, no differences were found in terms of the provenance of vegetables (see Supplementary Material Table A4). However, in both cases, the consumption of products of local origin exceeded 70% of preferences.

**Figure 4:**
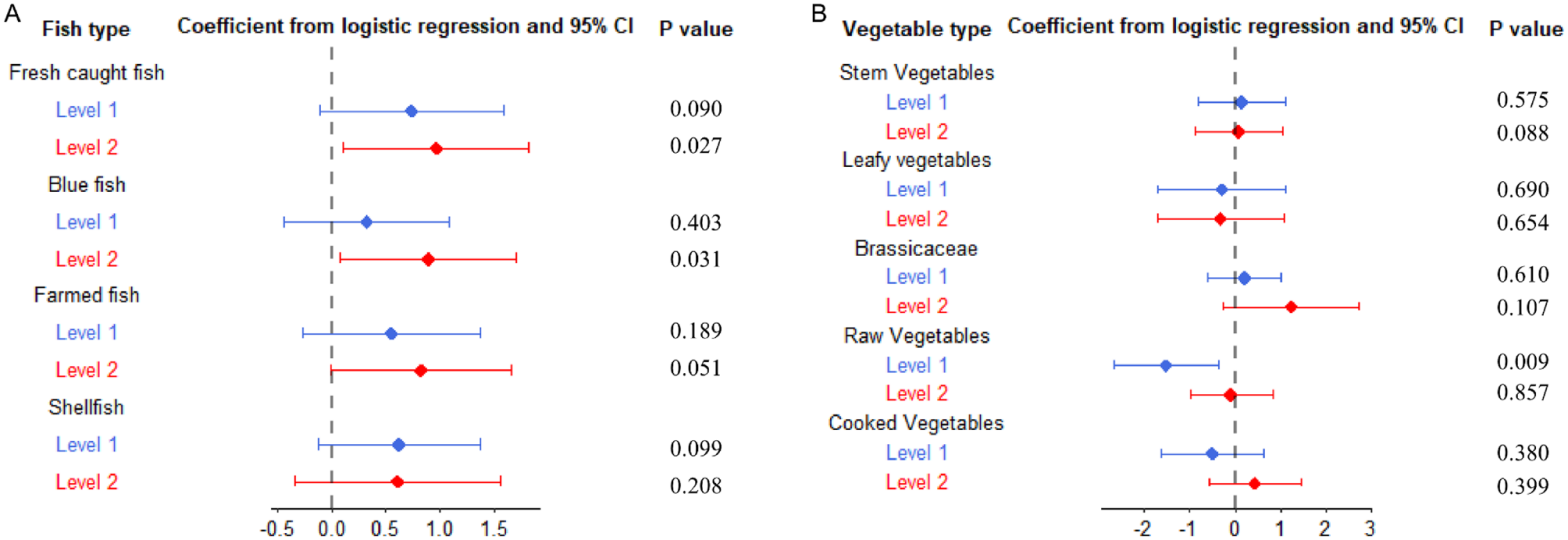
Forest plot relevant to the results of univariable logistic models built for each fish (**A**) and vegetable (**B**) categories. Each point shows the relevant estimate, horizontal bars refer to 95% confidence intervals.

Figure 4 shows the beta coefficients and their related confidence interval obtained from the univariable logistic models, for fish (Panel A) and vegetables (Panel B) items.

From the multivariable logistic model with a stepwise selection procedure, assessing the independent role of food categories in determining the clustering, “Blue fish” consumption was the only predictor retained in the final model (β = 0.91; ODDS = 2.49, p = 0.028).

The multivariable model for socio-demographic variables showed that only age (β = 0.25; ODDS = 1.29; p < 0.0001) and Area of residence (for LRA β = − 0.84; ODDS = 0.43; p = 0.022) were significant predictors. When all the considered variables were simultaneously included in a single multivariable model, the effect of the “blue fish” decreased and the variable was dropped out from the final model. The LASSO regression produced the same result: the only significant predictor was maternal age (β = 0.228, p < 0.0001).

We then tested the relationship between “blue fish” consumption and “age”: a significant association between the two variables was found, with older mothers consuming a greater amount of fish (p = 0.003). Moreover, in order to understand if fish consumption determined a major risk of belonging to H-Exp cluster independently from age, a subanalysis was performed on the subgroup of individuals (n = 22) who did not consume fish. The 68% (15/22) of mothers were in the L-Exp group, while 32% (7/22) were in the H-Exp. “Age” was not found to be significant in predicting clusters in a logistic univariable model in this subgroup.

Figure A2 in Supplementary Material reports the barplot showing the weights assigned to each variable ordered from higher to lower weights, as resulted from WQS model. The beta coefficient of the mixture was not significant and equal to 0.62 (95% CI including the zero: − 0.21/1.45). However, the results seem to indicate that “Shellfish” and “Blue Fish” are the greatest contributors to the mixture effect.

## Discussion

### Comparison of biomonitoring results with worldwide databases

The results of the k-means cluster analysis applied to the 161 pregnant women suggest that (i) maternal residence only partially explains the higher levels of contaminants in cluster comparisons; (ii) the “higher exposure cluster” is characterised by a relatively higher consumption of local fish; (iii) women in the high exposure group are significantly older and have a higher educational level than the low exposure group.

In general, the concentrations of organochlorine (OC) compounds measured in this study were lower than those reported in pregnant women from other European countries such as Poland^37^, Norway^38^, the Netherlands^39^, Denmark^40^ and Spain^41–43^. Similarly, a study conducted near the industrial area of Brescia, in northern Italy, reported higher OC levels in maternal serum^44^ than those found in this survey. Conversely, PCB concentrations were higher than those previously found in Japanese^45^, Canadian^46^ and U.S.^47–49^ studies. Moreover, the levels found in our sample were very close to those reported by the multicentre European birth cohort study HELIX^50^, based on data produced in six different European countries. To our knowledge, only two studies have reported concentrations of total Hg in the serum of pregnant women^51,52^. In particular, Yau et al. performed a case–control study to test the association between serum Hg levels and autism spectrum disorders, without documenting a meaningful association^51^. The second study was conducted in Croatia where Sekovanic et al. analysed Hg in serum from mothers living both in continental and coastal areas^52^. This latter study found significant differences in Hg concentration between the two areas but, in both cases, the levels of Hg reported in those studies were lower compared to our data. In 2011, Alimonti et al. piloted a wide biomonitoring survey in Italy, the PROBE study (PROgramme for Biomonitoring general population Exposure), which assessed the internal dosage of 20 metals in a representative sample of the Italian population^53^. The levels of Hg in the Italian female population were lower on average than those found in our sample, in particular of women residing inside the NPCS (arithmetic mean = 0.70 μg/L and 0.88 μg/L respectively). This also reflects the outcomes of previous investigations in the Priolo area^16,54^ and indicates a crucial exposure of the local population to Hg.

### Environmental contaminants and exposure pathways in the Priolo site

As shown in Table 1, no major differences in the socioeconomic variables were found between the mothers enrolled in NPCS and LRAs, with the exception of the educational level, which appears higher in the NPCS. The comparison between the serum levels of selected contaminants in mothers from the NPCS and LRAs (Table 2) shows a significantly higher concentration of HCB and PCBs in the NPCS group, while the higher concentration of Hg was not statistically different (p = 0.402). Remarkably, unlike other studies^55,56^, in our sample maternal Hg serum concentration was not associated with dental amalgam (p = 0.603—median and [IQR] of 0.60 µg/L [< LOQ-1.20 µg/L] and 0.66 µg/L [< LOQ-1.22 µg/L] for mothers without and with dental amalgam, respectively). In addition, although higher concentrations of the same group of measured contaminants were found in other studies, the exposure levels required for endocrine disruption during pregnancy are reported to be extremely low^57,58^. However, synergistic or additive effects between pollutants have been increasingly documented^59–61^. In light of this concern, we performed k-means cluster analysis aimed at identifying groups of mothers with different exposure levels. The two clusters show profiles of cumulative chemical exposure that might be associated with first-order indices of impact on health outcomes^62^. Using a k-means clustering algorithm, we identified two distinct clusters of women based on serum contaminant concentrations. Specifically, pollutant levels in mothers from the H-Exp group were at least twofold higher in concentration with respect to the L-Exp group (Table A3 in Supplementary Materials). The median values of HCB and PCBs found in the H-Exp group exceeded the values reported in the above-mentioned work of Montazeri et al., which reported serum OC levels from six birth cohorts of different European countries (HCB median values = 9.74 vs 8.20 ng/g; PCB138 = 11.66 vs 9.1 ng/g; PCB153 = 21.66 vs 17.6 ng/g and PCB180 = 16.26 vs 10.4 ng/g, respectively, for our sample and Montazeri et al.)^50^.

As mentioned above, the H-Exp group contained 61% of mothers living within the NPCS and 39% residing in the control area. This emphasises that higher contaminant levels can be found not only in individuals living in the NPCS, but also in the LRAs, and that pollutants may be rationally associated with common sources and pathways of contamination. Reasonably, local food and associated diets, reflecting the impact of environmental contamination, may represent a major pathway for transferring pollutants to humans, also for those populations living at a some distance from the emission site and primarily ‘linked’ to the same supply chains.

Such a ‘food hypothesis’ is also corroborated by evidence that mothers in the H-Exp group were characterised by significantly higher levels of fish consumption. Mean total fish consumption in the H-Exp group (929.4 g/month) is in line with a previous study of fish consumption in 17 European birth cohorts (plus one American), which found an overall mean consumption of 1.5 times/week, corresponding to about 900 g per month^63^. In the same study, the average consumption of fish for the Spanish birth cohort was 4.5 times/week, three times higher than those found in our cohort. These data could partially explain the higher levels of OCs found in their cohort than those found in our samples. The differences in fish consumption in the two clusters remain significant, even considering any individual fish category both in terms of grams/month (Fig. 3) and consumption frequency in univariable models (Fig. 4). Moreover, those in the cluster characterised by the highest exposure levels preferably consumed local fish (Supplementary Material Table A4). Traina et al. reported a systematic correlation of PCBs (considering the same congeners), HCB and Hg between benthic commercial fish and marine sediments in Augusta Bay, thus demonstrating a robust fingerprinting of contamination pathways^11^. This suggests a link between the highly polluted marine sediments of Augusta Bay that primarily drive benthic fish contamination, that, in turn, mirrors the higher levels of (analogue) contaminants in pregnant women with diets characterised by preference for local fish. In particular, among other fish categories, “Blue fish” was the only one variable retained in the multivariable model by the stepwise procedure.

Interestingly, we found that the H-Exp cluster was composed of a higher percentage of women with higher educational level: this result is in agreement with our recent study^27^ showing that, in pregnant women, higher educational stage and older age appear to enhance attention toward a “healthy” dietary pattern characterised by higher fish (bluefish in particular) and vegetable consumption. Nevertheless, a similar diet, in a highly contaminated area, could produce a counterintuitive effect with a higher risk for exposure to environmental pollutants. With regard to fish consumption and its origin, our results confirm the data of the numerous studies carried out in the same areas on the risk of consuming local fish severely impacted by polluted sediments.

Notably, the H-Exp group consisted mainly of older women, suggesting bioaccumulation effects for all the analysed pollutants^64–66^. We are aware of the difficulty to distinguish among age, bioaccumulation due to ageing, and the significant association found between age and blue fish consumption. In this regard, in order to assess if belonging to the high exposure cluster still depends on age in the subgroup of individuals who do not consume fish, we applied a logistic model to the 22 mothers with a free-fish diet. The beta coefficient of age from the logistic univariable model was not significant. This result, despite the small number of subjects, seems to suggest that the association between cluster and age is subordinated to the consumption of polluted food.

To our knowledge, this is the first biomonitoring study investigating serum levels of Hg and OCs in a sample of pregnant women residing in a NPCS. On our view, the present work has some limitations. The first is recruitment, performed on a voluntary basis, which could have been influenced by a similar sociocultural level of the participants, joined by a common interest toward the health-related aspects of living in highly polluted areas. Moreover, it remains difficult—mainly due to the small sample size—to disentangle the existing relationships among age, dietary pattern, socioeconomic status and exposure level. In fact, while age and socioeconomic status are able to influence dietary habits, age, per se, is a risk factor for bioaccumulation by several routes, including diet.

Despite these limitations, our findings highlight an urgent need to inform pregnant women living in highly contaminated areas about the risk arising from pollutants^67^, as well as to suggest healthy lifestyle habits and diets, even outside the pregnancy period. Remarkably, transfer routes of pollutants across the food chain and potentially reaching humans through daily diet appear priority areas of research. This should inspire and support urgent large scale studies to address possible interventions policies for mitigating environmental impact on highly sensitive subgroups of population.

### Supplementary Information

Supplementary Information.

## Data Availability

All relevant data are within the manuscript and Supplementary Material. Anonymized raw data are available upon request from the corresponding author.
