# Supplementary material for: Exposure profiles in pregnant women from a birth cohort in a highly contaminated area of southern Italy
Source: Sci Rep. 2023 Sep 8;13:14815. doi: 10.1038/s41598-023-41865-0 (PMC10491776; doi:10.1038/s41598-023-41865-0)
Supplement: Supplementary file 1 — Supplementary Information. [file 41598_2023_41865_MOESM1_ESM.docx]

**Exposure profiles in pregnant women from a birth cohort in a highly contaminated area of southern Italy**

Gaspare Drago^a^, Silvia Ruggieri^a*^, Mario Sprovieri^b^, Giulia Rizzo^a^, Paolo Colombo^a^, Cristina Giosuè^c^, Enza Quinci^d^, Anna Traina^c^, Amalia Gastaldelli^e^, Fabio Cibella^a^, Simona Panunzi^f^

^a^National Research Council of Italy, Institute for Biomedical Research and Innovation, via Ugo La Malfa 153, 90146 Palermo, Italy; gaspare.drago@irib.cnr.it (G.D.); silvia.ruggieri@irib.cnr.it (S.R.); giulia.rizzo.996@gmail.it (G.R.); paolo.colombo@irib.cnr.it (P.C.); fabio.cibella@irib.cnr.it (F.C.);

^b^National Research Council of Italy, Institute of Marine Sciences, Arsenale - Tesa 104, Castello 2737/F, 30122 Venice, Italy; mario.sprovieri@cnr.it (M.S.);

^c^National Research Council of Italy, Institute of Anthropic Impacts and Sustainability in Marine Environment, Lungomare Cristoforo Colombo 4521, 90149 Palermo, Italy; cristina.giosue@ias.cnr.it (C.G.); anna.traina@ias.cnr.it (A.T.)

^d^National Research Council of Italy, Institute of Anthropic Impacts and Sustainability in Marine Environment, via del Mare 3, 91021 Torretta Granitola, Trapani, Italy; enza.quinci@ias.cnr.it (E.Q.);

^e^National Research Council of Italy, Institute of Clinical Physiology, Via Giuseppe Moruzzi 1, 56124 Pisa, Italy; amalia@ifc.cnr.it

^f^National Research Council of Italy, Institute for System Analysis and Computer Science - BioMatLab, Via dei Taurini 19, 00168 Rome, Italy; simona.panunzi@biomatematica.it (S.P.)

^*^Corresponding author at: Istituto per la Ricerca e l'Innovazione Biomedica (IRIB), del Consiglio Nazionale delle Ricerche, Via Ugo La Malfa 153, 90146 Palermo, Italy. E-mail address: silvia.ruggieri@irib.cnr.it.

**Supplementary Material**

Figure A1: Illustration of methods used to identify the optimal number of clusters.

Figure A2: Estimated weight distribution from WQS model

Table A1: List of municipalities included in the study and their location with respect to industrial settlement.

Table A2: Comparison between the selected study sample and the whole birth cohort.

Table A3: Contaminant serum levels between the two identified clusters.

Table A4: Purchase origin of fish and vegetables and exposure cluster.

**Appendix A**

**
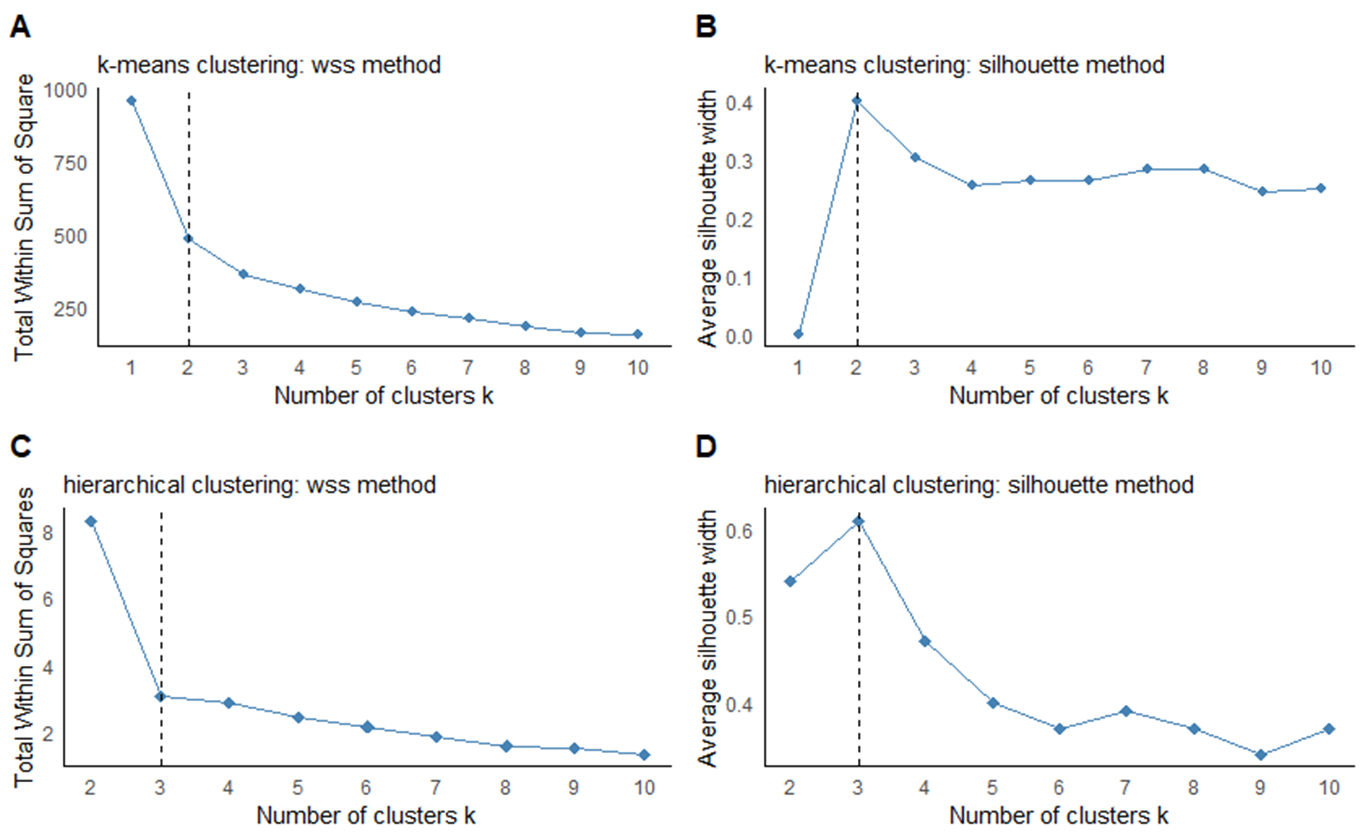
**

**Figure A1.** Elbow and Silhouette methods show the values of the two indices in correspondence to different Ks.


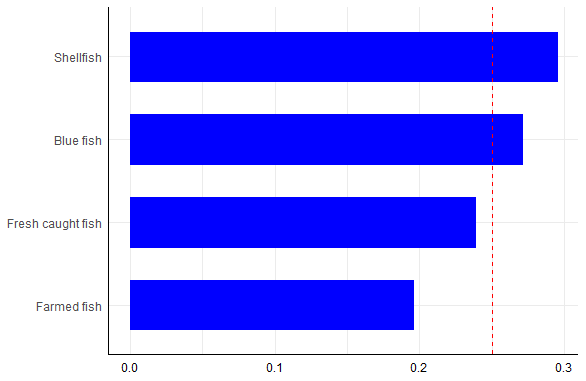


**Figure A2.** Estimated weight distribution for categories of fish consumption resulted from WQS model

**Table A1.** Municipalities selected for the project in both study and local reference areas, with their respective distance from industrial settlement.

| **National Priority Contaminated Site** | **Municipalities in the study area** | **Distance from industrial settlement (km)** | **Municipalities in the reference area** | **Distance from industrial settlement (km)** |
| --- | --- | --- | --- | --- |
| Priolo | Augusta  Priolo Gargallo  Melilli  Solarino  Floridia | 7  5  8  12  11 | Avola  Canicattini Bagni  Carlentini  Lentini  Noto  Pachino  Palazzolo Acreide  Rosolini  Francofonte  Palagonia  Scordia  Sortino  Vizzini | 27  19  22  22  45  59  51  57  49  61  50  25  63 |

**Table A2 –** Comparison of socio-demographic characteristics between the present study sample and the whole Priolo NEHO cohort.

|  | | | **Study sample (N = 161)** | | **NEHO Priolo cohort (N = 561)** | |  |
| --- | --- | --- | --- | --- | --- | --- | --- |
|  | | | **Mean** | **SD** | **Mean** | **SD** | **P value** |
| **Age (years)** | |  | 30.71 | 4.66 | 30.92 | 5.23 | 0.556* |
| **BMI (Kg/m^2^)** | |  | 23.24 | 4.78 | 23.60 | 4.85 | 0.341* |
| **Gestational length (weeks)** | |  | 39.04 | 1.22 | 39.41 | 1.23 | 0.754* |
|  | |  | **N Total** | **%** | **N** | **%** |  |
| **Educational level** |  | |  |  |  |  | 0.064# |
|  | Secondary school or lower qualification | | 30 | 18.63 % | 158 | 28.16% |  |
|  | High School | | 92 | 57.15 % | 277 | 49.38% |  |
|  | Degree or higher qualification | | 39 | 24.22 % | 124 | 22.10% |  |
|  | Missing | | - | - | 2 | 0.36% |  |
| **Marital status** |  | |  |  |  |  | 0.359# |
|  | Married | | 104 | 64.60 % | 341 | 60.80 % |  |
|  | Never married/Separated | | 55 | 34.16 % | 218 | 38.84 % |  |
|  | Missing | | 1 | 0.6 % | 2 | 0.36 % |  |
| **Previous pregnancy** |  | |  |  |  |  | **0.045**# |
|  | Nulliparous | | 77 | 47.82 % | 259 | 46.17 % |  |
|  | Parous | | 76 | 47.21 % | 172 | 30.66 % |  |
|  | Missing | | 8 | 4.97 % | 130 | 23.17 % |  |
| **Dental Amalgams** |  | |  |  |  |  | 0.524# |
|  | Yes | | 85 | 52.80 % | 281 | 50.10 % |  |
|  | No | | 69 | 42.86 % | 199 | 35.47 % |  |
|  | Missing | | 7 | 4.34 % | 81 | 14.43 % |  |

*SD: Standard Deviation; *p-value from Mann-Whitney U-test; # p-value from Chi-square test*

**Table A3 -** Contaminant serum levels between the two identified clusters. Serum levels of HCB and PCBs were normalized to total lipid content and reported in ng/g lipids.

| **Pollutant** | **Cluster 1**  **(N=84)** | | **Cluster 2**  **(N=77)** | | **P value*** | |  |
| --- | --- | --- | --- | --- | --- | --- | --- |
|  | **Median**  **(IQR)** | | **Median**  **(IQR)** | |  | |  |
| **Hg**  **(µg/L)** | | 0.42  (0.20-0.74) | | 0.96  (0.55-1.56) | | **< 0.0001** | |
| **HCB**  **(ng/g)** | | 5.83  (4.60-7.13) | | 9.74  (7.32-11.88) | | **< 0.0001** | |
| **PCB138**  **(ng/g)** | | 5.34  (4.17-6.97) | | 11.66  (9.57-15.73) | | **< 0.0001** | |
| **PCB153**  **(ng/g)** | | 9.41  (6.95-11.55) | | 21.66  (16.99-28.04) | | **< 0.0001** | |
| **PCB180**  **(ng/g)** | | 6.46  (4.59-7.64) | | 16.26  (12.50-21.21) | | **< 0.0001** | |
| **ΣPCB^a^**  **(ng/g)** | | 21.27  (15.90-26.25) | | 49.68  (40.51-64.49) | | **< 0.0001** | |

*IQR: Interquartile Range; *p-value from Mann-Whitney U-test for the differences between the two clusters; ΣPCB^a^: Sum of PCB138, PCB153 and PCB180 congeners; concentrations below the LOQ were replaced by LOQ/2 before lipid adjustment.*

**Table A4**. Percentages of purchase origin of fish and vegetables in relation to the k-means clusters.

|  |  | **Cluster 1**  **Low exposure** | **Cluster 2**  **High exposure** | **p value** |  |
| --- | --- | --- | --- | --- | --- |
| Fresh caught fish | *Large retail chains* | 20.31 | 8.47 | 0.047 |  |
|  | *Local distribution* | 79.69 | 91.52 |  |  |
| Blue fish | *Large retail chains* | 20.75 | 4.08 | 0.016 |  |
|  | *Local distribution* | 79.24 | 95.92 |  |  |
| Farmed fish | *Large retail chains* | 21.31 | 7.02 | 0.036 |  |
|  | *Local distribution* | 78.69 | 92.98 |  |  |
| Shellfish | *Large retail chains* | 15.15 | 17.86 | 1 |  |
|  | *Local distribution* | 84.85 | 82.14 |  |  |
| Stem vegetables | *Large retail chains* | 26.25 | 25.68 | 0.961 |  |
|  | *Local distribution* | 73.75 | 74.32 |  |  |
| Leafy vegetables | *Large retail chains* | 21.52 | 22.08 | 0.945 |  |
|  | *Local distribution* | 78.48 | 77.92 |  |  |
| Brassicaceae | *Large retail chains* | 16.67 | 22.39 | 0.261 |  |
|  | *Local distribution* | 83.33 | 77.61 |  |  |
| Raw vegetables | *Large retail chains* | 23.68 | 25.00 | 0.377 |  |
|  | *Local distribution* | 76.32 | 75.00 |  |  |
| Cooked vegetables | *Large retail chains* | 28.57 | 23.88 | 0.663 |  |
|  | *Local distribution* | 71.43 | 76.12 |  |  |

*p-values from Mann-Whitney U-test*
